# Whole genome sequencing and disease pattern in patients with juvenile polyposis syndrome: a nationwide study

**DOI:** 10.1007/s10689-023-00338-z

**Published:** 2023-06-24

**Authors:** Anne Marie Jelsig, Thomas van Overeem Hansen, Lene Bjerring Gede, Niels Qvist, Lise-Lotte Christensen, Charlotte Kvist Lautrup, Ken Ljungmann, Louise Torp Christensen, Karina Rønlund, Pernille Mathiesen Tørring, Birgitte Bertelsen, Lone Sunde, John Gásdal Karstensen

**Affiliations:** 1 Department of Clinical Genetics, University Hospital of Copenhagen, Rigshospitalet, Copenhagen, Denmark; 2 Department of Clinical Medicine, Faculty of Health and Medical Sciences, University of Copenhagen, Copenhagen, Denmark; 3 Research Unit for Surgery, Odense University Hospital, Odense, Denmark; 4 University of Southern Denmark, Odense, Denmark; 5 Department of Molecular Medicine, University Hospital of Aarhus, Aarhus, Denmark; 6 Department of Clinical Genetics, Aarhus University Hospital, Aarhus, Denmark; 7 Department of Surgery, Aarhus University Hospital, Aarhus, Denmark; 8 Department of Surgery, Slagelse, University Hospital of Zealand, Slagelse, Denmark; 9 Department of Clinical Genetics, University Hospital of Southern Denmark, Vejle Hospital, Vejle, Denmark; 10 Department of Clinical Genetics, Odense University Hospital, Odense, Denmark; 11 Center for Genomic Medicine, University Hospital of Copenhagen, Rigshospitalet, Copenhagen, Denmark; 12 Department of Clinical Genetics, Aalborg University Hospital, Aalborg, Denmark; 13 Department of Clinical Medicine, Aalborg University, Aalborg, Denmark; 14 Danish Polyposis Registry, Gastrounit, Copenhagen University Hospital – Amager and Hvidovre, Hvidovre, Denmark; 15 Department of Clinical Medicine, University of Copenhagen, Copenhagen, Denmark

**Keywords:** Juvenile, Polyp, Cancer, Syndrome, Hereditary

## Abstract

Juvenile polyposis syndrome (JPS) is a hereditary hamartomatous polyposis syndrome characterized by gastrointestinal juvenile polyps and increased risk of gastrointestinal cancer. Germline pathogenic variants are detected in *SMAD4* or *BMPR1A*, however in a significant number of patients with JPS, the etiology is unknown. From Danish registers, and genetic department and laboratories, we identified all patients in Denmark with a clinical diagnosis of JPS and/or a pathogenic variant in *BMPR1A* or *SMAD4*. In patients where no variant had been detected, we performed genetic analysis, including whole genome sequencing. We collected clinical information on all patients to investigate the phenotypic spectrum. Sixty-six patients (mean age 40 years) were included of whom the pathogenic variant was unknown in seven patients. We detected a pathogenic variant in *SMAD4* or *PTEN* in additional three patients and thus ≈ 95% of patients had a pathogenic germline variant. Endoscopic information was available in fifty-two patients (79%) and of these 31 (60%) fulfilled the clinical criteria of JPS. In 41 patients (79%), other types of polyps than juvenile had been removed. Our results suggest that almost all patients with a clinical diagnosis of JPS has a pathogenic variant in mainly *BMPR1A*, *SMAD4*, and more rarely *PTEN*. However, not all patients with a pathogenic variant fulfil the clinical criteria of JPS. We also demonstrated a wide clinical spectrum, and that the histopathology of removed polyps varied.

**Supplementary Information:**

The online version contains supplementary material available at 10.1007/s10689-023-00338-z.

## Introduction

Juvenile polyposis syndrome (JPS) is a hereditary hamartomatous polyposis syndrome. The incidence has been estimated to 1:100,000–160,000 [1]. The syndrome is characterized by gastrointestinal juvenile polyps (JPs), mainly the colon, rectum, and stomach as well as an increased risk of colorectal (CRC) and gastric cancer (GC) [2, 3]. The inheritance pattern is autosomal dominant, and symptoms typically develops in adolescence.

The diagnosis is clinical and based on the Jass criteria, where one or more of the following must be fulfilled: (1) more than five juvenile polyps of the colorectum, (2) multiple juvenile polyps throughout the gastrointestinal tract and/or (3) any number of juvenile polyps with a family history of JPS [4].

In four studies from 2007 to 2021, a pathogenic germline variant was identified in *SMAD4* or *BMPR1A* in 45–60% of patients fulfilling the criteria of JPS, suggesting that variants in other genes may be causative [1, 5–8]. However, in the study by Latchford published in 2012, the detection rate was 82%, leaving open the possibility that the lower detection rate in other studies have other causes [1]. The identification of the predisposing gene variant is important as surveillance should be tailored based on genotype and because patients can have the possibility of prenatal diagnostics including pre-implantation genetic diagnostics.

Patients with a pathogenic variant in *SMAD4* (*SMAD4*-related JPS) often present with an additional phenotype not seen in patients with a pathogenic variant in *BMPR1A* (*BMPR1A*-related JPS) or in patients with JPS with unknown etiology [9]. This additional phenotype includes symptoms of hereditary hemorrhagic telangiectasia (HHT) (recurrent epistaxis, AV-malformations primarily in the lungs, liver and brain, besides skin/mucosal telangiectasias) [10]. The patients also have an increased risk of aortic root dilatation [11, 12]. Additionally, patients with *SMAD4*-related JPS have a more severe gastric phenotype including increased risk of massive polyposis, GC, and characteristic endoscopic features of the gastric mucosa [13].

In this study, we included patients that fulfilled the clinical criteria of JPS and/or had a pathogenic variant in *SMAD4* or *BMPR1A*. We performed genetic analysis, including WGS, to detect the genetic etiology in as many patients as possible. Furthermore, we also collected clinical information to describe the phenotypic spectrum of the syndrome.

## Materials and methods

### Identification of patients

Patients were identified from The Danish Pathology Register; a search was performed using the Danish version of the Systematized Nomenclature of Medicine (SNOMED) diagnostic codes for “hamartomatous polyp” and “juvenile polyp.”

In addition, all Danish genetic departments and laboratories were asked to identify patients with a clinical diagnosis of JPS and/or a pathogenic variant (PV) in *BMPR1A* or *SMAD4.* A variant was classified as a PV if it was classified as “pathogenic” or “likely pathogenic” according to the guidelines of American College of Medical Genetics (ACMG) [14]. Information on family members were also collected, and relatives with signs or symptoms of JPS (early CRC/GC, juvenile polyps and/or HHT symptoms) were included.

The study was approved by The Danish Patient Safety Authority (journal no. 31-1521-329), the Regional Danish Data Protection Agency (journal-no.: P-2020-557/P-2020-696, The Capital Region of Denmark and the National Scientific and regional Scientific Ethics Committee (no-2105809/H-16030776).

### Inclusion

A patient was included if the Jass criteria were fulfilled and/or if the patient were heterozygous for a PV in *BMPR1A* or *SMAD4.* Patients at all ages as well as deceased patients were included.

*Genetic analysis* If the etiology was not known, patients were contacted and offered genetic counselling and (re-)testing. Genetic analyses were performed on DNA extracted from peripheral blood. Primarily, a custom-made gene panel including *BMPR1A, PTEN* and *SMAD4*, as well as other genes associated with an increased risk of polyposis and colorectal cancer (*APC*, *AXIN2*, *GREM1*, *MLH1, MSH2, MLH3*, *MSH6*, *MUTYH*, *NTHL1*, *PMS2, POLD1*, *POLE*, *STK11*), was analyzed by NGS (Illumina Technology). The sequencing analysis enabled detection of single nucleotide variants in the coding regions as well as in the first and last 50 bp of the intronic regions, along with detection of copy number variants (CNVs). If a PV was not revealed, WGS was performed with Illumina Technology and sequencing to a median depth of at least 30×.

*Clinical data* Clinical information on all patients, both deceased and alive, was retrieved from Danish registers and medical records throughout the country. Data were collected from relevant departments, including surgical, pediatric, and oncological departments.

*Study period* Information on each person was retrieved from birth to death or until December 31st, 2021.

*Statistics* The point prevalence for 2021 was calculated based on the total Danish population retrieved from Statistics Denmark (5,843,347 residents) pr. 31st of December 2021. Descriptive data are presented in absolute numbers and proportions (%). Probabilities of cancer were estimated with Kaplan–Meier analysis. For patients who had been diagnosed with cancer, the time to event was not truncated at any age. For cancer probability, the follow-up time in the model were the time between date of birth and 31.12.2021, date of death, date of loss of follow up or date of first cancer diagnosis, whichever came first. The statistical software R version 4.2.1 (R Foundation for Statistical Computing, Vienna, Austria) was used in the analysis.

## Results

**Fig. 1:**
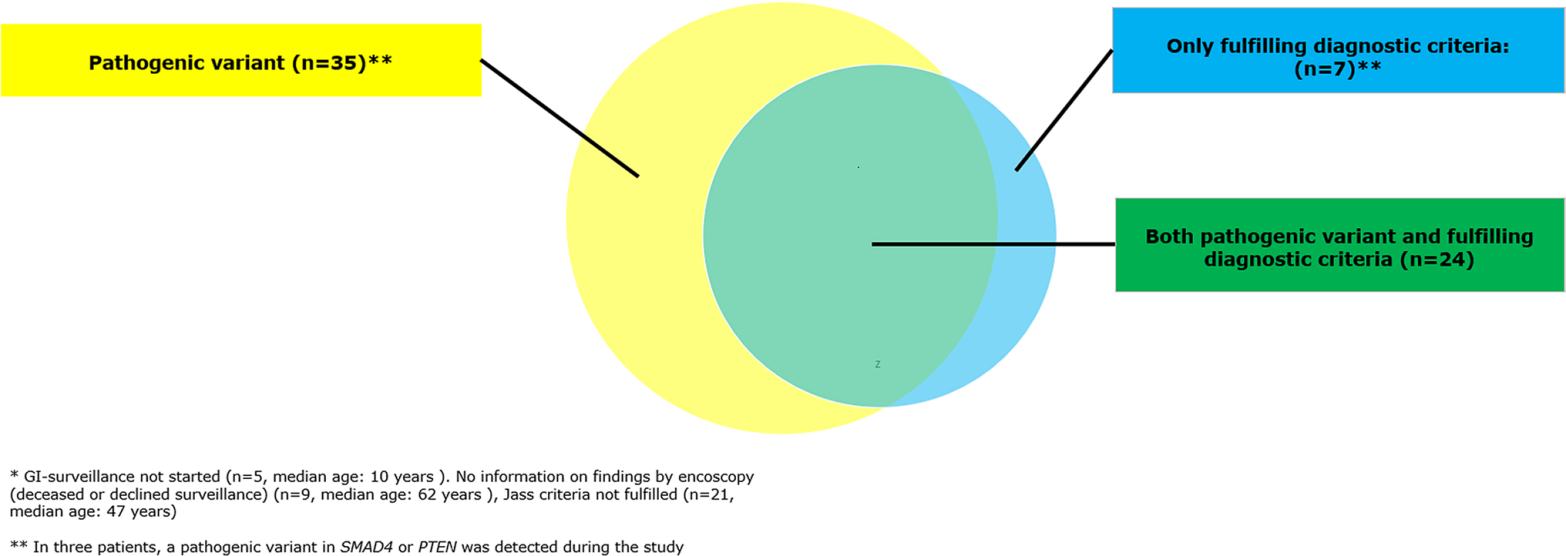
Patients carrying a pathogenic variant and/or fulfilling clinical criteria at study inclusion (n = 66)

Sixty-six patients (34 males) fulfilled the inclusion criteria. Seven patients were included based on a clinical diagnosis of JPS, only, while a PV had been detected in *BMPR1A* or *SMAD4* in 59 patients (Fig. 1) of which 24 patients also fulfilled the clinical criteria. Thereby, 35 patients were included based on a detected PV alone. These patients had been genetically tested because a PV had been detected in a family member. Average age at the time of study was 40 years. Thirteen patients were deceased. Five patients were under the age of 12 years, where GI-surveillance begins, and 9 patients had not had a GI-endoscopy—either because they were diagnosed posthumously or because they declined surveillance. The estimated point prevalence of patients being heterozygous for a PV in *SMAD4* or *BMPR1A* and/or a clinical diagnosis of JPS was 1:110.000.

### Genetic analysis

In seven non-related patients, no PV had previously been identified. These patients were all identified in The Danish Pathology Register, and they all fulfilled the first Jass criterion (more than 5 juvenile colorectal polyps).

All seven patients were contacted. One patient did not respond to our inquiry, while the remaining six patients consented participation. By analyzing the NGS panel of polyposis associated genes, two different PVs in *SMAD4* were detected in the two patients who did not follow JPS-surveillance, while it was not possible to detect a PV in the remaining four patients. WGS was then performed, and in one of these an intronic pathogenic variant in *PTEN* was detected. The patient was diagnosed with macrocephaly and multinodular non-toxic goiter; however, he did not fulfill the clinical criteria of Cowden syndrome. He had multiple colorectal polyps removed since the age 33. The polyps were mainly described as hamartomatous juvenile polyps, but in some cases, they were difficult to distinguish from an inflammatory polyp. Therefor he fulfilled the clinical criteria of JPS.

In three other patients, no PV or variant(s) of unknown significance were detected despite meticulous analysis of polyposis-associated genes, including *BMPR1A*, *SMAD4,* and *PTEN*. One of these patients had six juvenile polyps removed at 11–13 years of age and had not had any other polyps detected since (last follow-up at 25 years of age). The two other patients both had app. 10–20 colonic juvenile polyps removed and one was also diagnosed with gastric and duodenal hamartomatous polyps and had a history of epistaxis. None of these three patients had a relative with JPS. We observed no indication of mosaicism, as DNA from polyp tissue were analyzed with the NGS panel in two of these patients. In the third patient the quality of DNA in the polyp was not sufficient for genetic analysis.

**Fig. 2:**
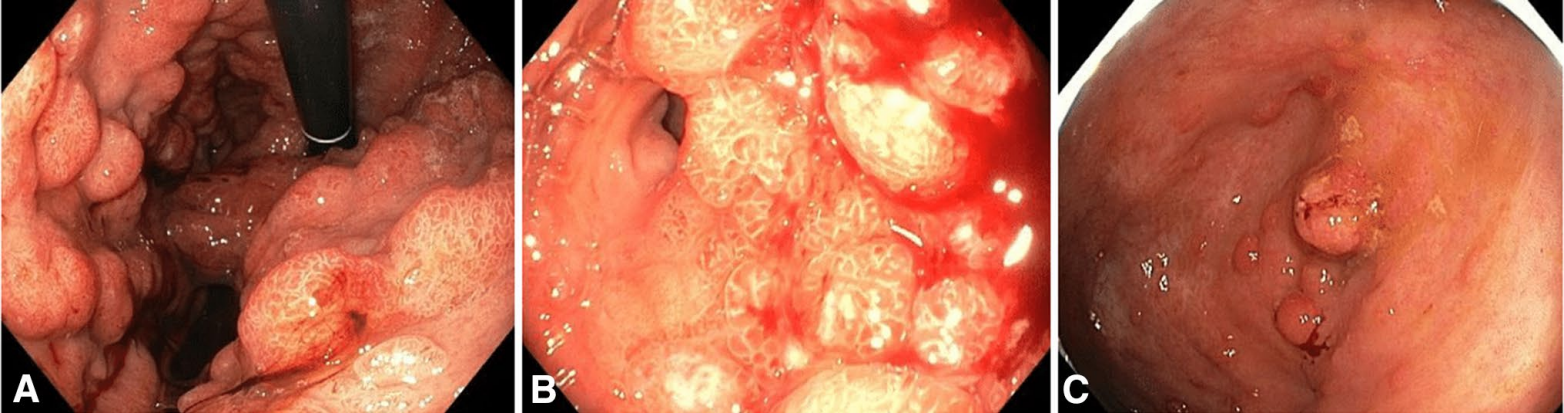
Endoscopic pictures of polyps in patients with Juvenile Polyposis Syndrome and pathogenic variants in *SMAD4*. **A**, **B** upper GI-tract, **C** colon

Both patients in whom we detected a PV in *SMAD4* had 10–20 colonic polyps removed over a period of 15–20 years, that had shown inflammatory or hyperplastic histopathology with only few polyps suspected to be juvenile. Upon detection of the PVs in *SMAD4* they were recommended additional upper GI surveillance. Both were then diagnosed with massive polyposis (Fig. 2) and subsequently had a gastrectomy. A summary of the patients included, and the genetic findings is presented in Table 1.

**Table 1:** Information on patients and families, at the end of the study, stratified by genetic findings

	No PV	*BMPR1A*	*SMAD4*	*PTEN*	Total
Number of patients (male:female)	4* (2:2)	20 (10:10)	41 (21:20)	1 (1:0)	**66 (34:32)**
Number of families	4*	7	15	1	**27**
Mean age	34 years	48 years (11–73 years)	40 years (2–78 years)	59 years	**40 years (2–78 years)**
Deceased patients	0	4	9	0	**13**
Mean age at death	–	59 years (37–86 years)	54 years (25–77 years)	–	**57 years**
Family history: yes/no/unknown	0/4/0	15/2/4	30/7/4	0/1/0	45/13/8

*In one patient/family genetic analyses were not performed

### Genotype

Among the 65 patients where genetic analysis was performed, a PV was detected in *BMPR1A, SMAD4* and/or *PTEN* in 62 patients (95%). One patient had a deletion of both *BMPR1A* and *PTEN,* and one patient had a PV in *PTEN*, only. In the 3 patients who fulfilled the clinical criteria and in which a blood sample had been subjected to genetic analyses including WGS, no relevant variant was detected. The gene variants detected are given in supplementary Table 1.

### GI-manifestations

Information on GI-manifestations in patients who had endoscopic examinations (n = 52) is described in Table 2. Thirty-one patients (60%) fulfilled the clinical criteria of JPS with over five juvenile polyps in the GI-tract, and of these a PV could be found in 27 (87%)*.* All 52 patients had one or more polyps removed. About 20% received a hemicolectomy or colectomy either because of massive polyposis or because of CRC. Gastrectomy was performed in 14% of the patients because of massive polyposis or GC—all of these had a PV in *SMAD4.*

**Table 2:** Manifestations of the GI tract

	No PV	*BMPR1A*	*SMAD4*	*PTEN*	Total
Fulfilling clinical criteria (more than five colorectal juvenile polyps)	4 (100%)	7 (50%)	19 (58%)	1	31 (60%)
Patients without any juvenile polyps	0	3 (21%)	4 (9%)	0	7 (13%)
Patient with adenomas, hyperplastic or inflammatory polyps in addition to juvenile polyps	3 (75%)	10 (71%)	28 (88%)	1	41 (79%)
Polyposis					
Gastric	1 (25%)	4 (29%)	29 (70%)	0	34 (65%)
Colorectal					
< 20 polyps	3 (75%)	6 (38%)	17 (52%)	1	25 (48%)
20–100 polyps	1 (25%)	6 (43%)	10 (30%)		17 (33%)
> 100 polyps	0	2 (14%)	6 (18%)		8 (15%)
Surgery					
Gastrectomy	0	0	9	0	9 (14%)*
Colectomy or hemicolectomy	0	4	10	0	14 (21%)*
Other	Resection of sigmoid colon	–	–	–	

*Percent of the total cohort

Table 2 shows that 41 patients (79%) on whom endoscopic information was available, had other types of polyps than juvenile removed. These included adenomas, hyperplastic and/or inflammatory polyps. Seven patients (13%) had not had any juvenile polyps removed but other types of polyps (mean age at end of follow-up: 47 years). The number of polyps varied between families, but also within families. Dysplasia in the juvenile polyp were detected in a minority of patients (under 10) and included dysplasia in both colonic and gastric polyps, However, no juvenile polyps were found with malignant alterations.

### Genotype–phenotype correlation

Most patients with *SMAD4*-related JPS had HHT-manifestations including epistaxis, telangiectasias and AV-malformations (mainly pulmonary), this was not noted in patients with PV in *BMPR1A* and/or *PTEN* (see Supplementary Table 2). Some patients with PV in *BMPR1A* had gastric polyps. However, gastric involvement was more frequent and more massive in patients with *SMAD4*-related JPS.

### Cancer

Seventeen patients (26%) had been diagnosed with cancer as presented in Table 3. The mean age at diagnosis was 48 years of age, however the age at diagnosis varied from 20 to 72 years. GC was diagnosed in two patients—both with *SMAD4*-related JPS—and both under 50 years at diagnosis. Pancreatic and oesophageal cancer were seen in patients with pathogenic *BMPR1A* variants. No cases of small bowel cancer were noted.

**Table 3:** Cancer occurrence

	No PV	*BMPR1A*	*SMAD4*	Total
Total number of cancers	0	5 (24%)	12 (29%)	17 (26%)
Average age all cancers	–	49 years (31–64 years)	47 years (20–72 years)	48 years (20–72 years)
Type of cancer				
Colorectal	–	3 (14%)	7 (11%)	10 (15%)
Gastric	–	0	2 (5%)	2 (3%)
Small bowel	–	0	0	0
Pancreatic	–	1 (5%)	0	1 (2%)
Other	–	1 (5%, Oesophageal)	3 (7%, lung, lymphoma)	4 (6%)

The probability of cancer was calculated over a total of 2782 person years. The cumulative probability of cancer at the age of 40 years was 12.6% [95% CI (3.1–22.2)], and at age 70 years 49.2% (95% CI 28.4–77.1). The probability of cancer tended to be higher in men [63%, 95% CI (25.0–100.0)], than female [43%, 95% CI (28.4–77.1)] at age 70 years.

## Discussion

This study is a nationwide study including all 66 Danish patients who fulfilled the clinical criteria of JPS and/or had a PV in *SMAD4* or *BMPR1A*. We found that almost 90% of patients with a clinical diagnosis of JPS had a PV in either *BMPR1A* or *SMAD4*. Endoscopy had been performed in 52 patients, and 21 (40%) of these did not fulfill the Jass criteria. Furthermore, the removed polyps frequently were other types than juvenile.

### SMAD4 and BMPR1A: the only genes associated with JPS?

Previous studies have reported a relatively low variant detection rate when analyzing *SMAD4* or *BMPR1A* indicating genetic heterogeneity [1, 5–8] (see Table 4). Our study as well as others suggest that PVs in *PTEN* are found in an additional (small) number of patients [7, 15]. The implementation of NGS, including WGS, did not add significantly to our detection rate.

**Table 4:** Detection of gene variants in JPS

Study	Number of patients/families fulfilling clinical Jass criteria	Technique	Genes investigated	Variant detection rate in *SMAD4* or *BMPR1A* (per family)	*BMPR1A*	*SMAD4*	*PTEN*	*BMPR1A/PTEN* deletion
Aretz et al. [7]	65/65	SS + MLPA	PTEN*, BMPR1A, SMAD4,	39 (60%)	16 (25%)	23 (35%)	2 (5%)	–
Van Hattem [8]	29/27	SS + MLPA	*PTEN, BMPR1A, SMAD4*, *ENG*	13/27 (48%)	4 (15%)	7 (26%)	–	2 (7%)
Calva-Cerqueira [5]	102/102	SS + MLPA	*BMPR1A, SMAD4*	45 (45%)	22 (23%)	22 (22%)	–	1 (1%)
Latchford et al. [1]	31/17	NR	NR	14 (82%)	9 (29%)	19 (61%)	–	–
MacFarland [6]	118/?	Sequencing and CNV analysis	*BMPR1A, SMAD4*	54 (46%)**	24 (20%)	27 (23%)	–	3 (3%)
Present study	31/27	NGS panel + WGS	*See text*	23 (85%)	6 (19%)	19 (61%)	1 (3%)	1 (3%)

*SS* Sanger sequencing, *NR* not reported

*In 60% of the patients, *PTEN* was not analyzed

**Per patient

The reason for the discrepancy in the variant detection rate between our study and most others is uncertain. Possibly, it could be caused by differences in methods used for identification and inclusion of patients. The mode of inclusion in our study is quite robust, as inclusion was based on data from The Danish Pathology Register: The register comprises data from all histopathological examinations carried out in Denmark since 1997, and for some departments even earlier. Although the significantly higher detection rate is unexplained, our findings, along with the findings by Latchford et al. suggest that in most cases, PJS is caused by a PV in either *SMAD4* or *BMPR1A.*

We observed no indication of mosaicism, and to our knowledge this has neither been reported in other studies. Yet, mosaicism for variants in *APC* and *STK11* has been reported [16–18], and it seems unlikely that mosaicism do not occur in patients with JPS. Possibly the phenotype in patients being mosaic for a variant in *SMAD4* or *BMPR1A* is so “mild” that it typically is overlooked.

### Varying histopathology leads to underdiagnosis

The high variant detection rate indicates that the Jass criteria [4] are a strong indicator of a pathogenic variant. However, our findings also suggest that there is a risk of missing patients with PVs in *SMAD4* or *BMPR1A* if using these criteria alone: only 60% of the patients with a PV in one of these genes, who had endoscopic investigations, fulfilled the Jass criteria. Thus, one can only speculate that JPS in general is underdiagnosed, and that the calculated incidence is an underestimation. This also suggests that the threshold for performing genetic analysis should be rather low.

The histopathology of the colorectal polyps varied greatly—an observation that has also been recognized in other studies although at a smaller scale [15, 19, 20]. All patients with a PV in *SMAD4* or *BMPR1A* had polyps removed and, furthermore, in approximately 15% of the patients no juvenile polyps (but other types) had been removed. This emphasizes that there should be an awareness, in the clinical setting, that JPS can present with a broad spectrum of polyps. This also indicates that *SMAD4* and *BMPR1A* should be included in the panel of genes analyzed in all patients suspected to suffer from a genetic predisposition to intestinal polyposis. However, the varying histopathology can also be due to misclassification of polyps in the first place as have been reported before [7, 21]. In general, it can be difficult to distinguish different types of polyps from each other and especially juvenile polyps can resemble inflammatory polyps.

The consequences of the varying histopathology were evident in the two patients who had 10–20 colonic polyps removed over a period of 15–20 years. The polyps had shown various histopathology with only few polyps suspected to be juvenile. When participating in our study, both patients were found to have a PV in *SMAD4* and were recommended additional upper GI surveillance. Both were then diagnosed with massive gastric polyposis and subsequently had a gastrectomy.

### Wide clinical spectrum leading to suboptimal surveillance

In general, we observed that the clinical spectrum of patients with a PV in *SMAD4* or *BMPR1A* was very wide: from massive polyposis (both gastric and colorectal) to very few colorectal polyps. Our findings also support the general conception of a difference in the phenotype of *SMAD4*-related JPS and *BMPR1A*-related JPS: We diagnosed more massive gastric involvement and HHT-manifestations in *SMAD4*-related JPS (Supplementary table 2) compared to *BMPR1A*-related JPS.

The highly variable phenotype suggests that the current surveillance guidelines impose a risk of overtreatment. According to most guidelines, surveillance of the GI tract should begin at age 12–15 years and examinations should be repeated every 2–3 years [22, 23]. Most agree that surveillance in JPS patients is indicated to reduce the risk of getting and dying from cancer as well as to reduce the morbidity caused by polyposis. However, there is a lack of long-term studies that can support the effect of surveillance. We calculated a probability of cancer at age 70 years to be approximately 50%, but various estimates have been reported and basically, a lack of knowledge concerning the pathophysiological mechanisms makes it difficult to tailor optimal surveillance [24–27]. As it is, a “one-size-fits-all” program does not seem to fit all. Possibly one could achieve a better balance between the “costs” (i.e. inconvenience of attending surveillance and the risk of overtreatment) and benefits for the individual patient by adjusting the surveillance program by the gene variant causing the predisposition and the personal history of the patient.

### Strength and limitations of the study

A limitation of this study is the relatively low number of patients included and a risk of overlooking patients with a mild phenotype. Also, the facts that we ascertained from various sources, that we ascertained both on phenotype and on genotype, and that the patients had various ages at the end of follow-up, may have limited the generalizability of our observations.

However, it is a major strength that our study is nationwide and conducted in Denmark, where the health care system offers services, without out-of-the pocket expenses, to all citizens, where all citizens are identifiable by the unique social security number, and where registration in nation-wide registers, such as the Danish Pathology Register, is mandatory. This made it possible to identify the patient’s medical data and histopathological samples across registers and departments throughout the country, several years back.

## Conclusion

In most patients, JPS is caused by a PV in *BMPR1A* or *SMAD4*, and the threshold for genetic analysis should be lowered. Not all patients with a PV in *BMPR1A* or *SMAD4* fulfil the Jass-criteria, mainly because polyps show various histopathology. Consequently, the incidence of JPS is probably underestimated. Long-term studies and further investigations into the underlying molecular mechanism are needed to improve surveillance strategies.

### Supplementary Information

Below is the link to the electronic supplementary material.

Supplementary file1 (DOCX 14 KB)

Supplementary file2 (DOCX 19 KB)
