# Supplementary material for: Whole genome sequencing and disease pattern in patients with juvenile polyposis syndrome: a nationwide study
Source: Fam Cancer. 2023 Jun 24;22(4):429–36. doi: 10.1007/s10689-023-00338-z (PMC10542306; doi:10.1007/s10689-023-00338-z)
Supplement: Supplementary file 1 — Supplementary file1 (DOCX 14 KB) [file 10689_2023_338_MOESM1_ESM.docx]

|  | **No PV** | ***BMPR1A*** | ***SMAD4*** |
| --- | --- | --- | --- |
| **HHT** |  |  |  |
| AV-malformations | NI | NI | 16 (59%) |
| Epistaxis | 1 (25%) | NI | 25 (86%) |
| Telangiectasias | NI | NI | 24 (83%) |
| Fulfilling Curacao criteria | NI | NI | 22 (76%) |
| Aortic abnormalities |  |  | 3 (5%) |

HHT manifestations

NI=not investigated
