# Supplementary material for: Whole genome sequencing and disease pattern in patients with juvenile polyposis syndrome: a nationwide study
Source: Fam Cancer. 2023 Jun 24;22(4):429–36. doi: 10.1007/s10689-023-00338-z (PMC10542306; doi:10.1007/s10689-023-00338-z)
Supplement: Supplementary file 2 — Supplementary file2 (DOCX 19 KB) [file 10689_2023_338_MOESM2_ESM.docx]

**Supplementary Table 1**

Pathogenic or likely pathogenic variants detected in the study

| **Exon number** | **Variant** | | **Type** |  |
| --- | --- | --- | --- | --- |
| ***BMPR1A*** (NM_004329.3) | | | | |
| Exon 4-5 | c.(67+1_68-1)_(333+1_334-1)del | p.(Gln23Phefs*19) | Frameshift |  |
| Exon 6 | c.355C>T | p.(Arg119Cys) | Missense |  |
| Exon 7 | c.454C>T | p.(Arg152Ter) | Nonsense |  |
| Exon 9 | c.826_827del | p.(Glu276Asnfs*10) | Frameshift |  |
| Intron 9 | c.868+2T>C | p.(?) | Splice site |  |
| Exon 10 | c.977dup | p.(Asp327Glyfs*7) | Frameshift |  |
|  |  |  |  |  |
| Exon 1-13 | Whole gene deletion of *BMPR1A* and *PTEN* |  | Large CNV |  |
| ***SMAD4*** (NM_005359.5) | | | | |
| Exon 3 | c.330dup | p.(His111Thrfs*3) | Frameshift |  |
| Exon 6 | c.692dup | p.(Ser232Glnfs*3) | Frameshift |  |
| Exon 6 | c.723_730del | p.(Gly243Alafs*18) | Frameshift |  |
| Exon 7 | c.831_832del | p.(Pro278Ter) | Nonsense |  |
| Exon 8 | c.939del | p.(Ile314Phefs*22) | Frameshift |  |
| Exon 9-10 | c.(955+1_956-1)_(1308+1_1309-1)del | p.(Ala319Glyfs*3) | Frameshift |  |
| Exon 9 | c.1081C>T | p.(Arg361Cys) | Missense |  |
| Exon 10 | c.1156G>A | p.(Gly386Ser) | Missense |  |
| Exon 10 | c.1245_1248del | p.(Asp415Glufs*20) | Frameshift |  |
| Exon 11 | c.1325_1326del | p.(Gln442Leufs*51) | Frameshift |  |
| Exon 11 | c.1421del | p.(Ser474Ter) | Nonsense |  |
| Intron 11 | c.1448-1G>A | p.(?) | Splice site |  |
| Exon 12 | c.1587dup | p.(His530Thrfs*47) | Frameshift |  |
|  | t(1;18)(p36.1;q21.1) |  | Chromosomal translocation |  |
| ***PTEN*** (NM_000314.8) | | | | |
| Intron 3 | c.209+2047A>G | p.(?) | Splice site |  |
